# Evaluation of the diagnostic accuracy of nonstructural protein 1 Ag-based tests for dengue virus in Asian population: a meta-analysis

**DOI:** 10.1186/s12879-015-1088-4

**Published:** 2015-08-21

**Authors:** Xiaoyun Shan, Xiangmei Wang, Qing Yuan, Yaping Zheng, Honghe Zhang, Yihua Wu, Jun Yang

**Affiliations:** Department of Clinical Laboratory, Jin Hua Hospital, Zhejiang University, 351 Ming Yue Street, Jin Hua, 321000, Zhejiang China; Collaborative Innovation Center for Diagnosis and Treatment of Infectious Diseases, The First Affiliated Hospital, Zhejiang University School of Medicine, 79 Qing ChunRoad, Hangzhou, 310003, Zhejiang China; Department of Public Health, Hangzhou Normal University School of Medicine, 16 Xuelin Street, Hangzhou, 310016 Zhejiang China

## Abstract

**Background:**

Nonstructural protein 1 (NS1) Ag-based tests are useful for detecting dengue virus (DENV), but there is lack of evidence on the diagnostic accuracy of NS1 Ag-based tests in Asian population. Thus, we conducted this meta-analysis to obtain the overall estimated and summarized performance of the NS1 Ag-based tests in the detection of DENV in Asia.

**Methods:**

PubMed, Embase and Medline were searched for studies that evaluated the diagnostic validity of NS1 Ag-based tests between January 1990 and November 2014. Data were analyzed by Meta-Disc and STATA software.

**Results:**

A total of 18 studies including 3342 dengue cases and 1904 control cases which fulfilled the inclusion criteria were considered for analysis. The pooled sensitivity and specificity for NS1 Ag-based tests was 66 % (95 % CI 64.5–67.5) and 97.9 % (95 % CI 97.3–100), respectively. STRIP has the overall highest sensitivity (72.9 %, 95 % CI 70.1–75.5). According to viral serotype, the test with the highest sensitivity for DENV1, DENV2 and DENV3 were Platelia (83.7 %, 95 % CI 79.7–87.1), Panbio (71.8 %, 95 % CI 65.5–80.9) and STRIP (81.9 %, 95 % CI 75.5–87.2) respectively. The highest sensitivity for primary infection was Platelia (95.1 %, 95 % CI 92.6–96.9) and for secondary infection was STRIP (64 %, 95 % CI 53.2–73.9).

**Conclusion:**

Our meta-analysis suggests that NS1 Ag-based test is a good diagnostic method for DENV with a high specificity. However, viral serotype, serological status, clinical severity and the duration of illness are the main factors influencing the diagnostic accuracy.

## Backgroud

Dengue, a vector-borne disease, has become one of the most serious public problems due to the increasing morbidity, with over one billion people at risk in tropical and subtropical areas [1]. Dengue virus (DENV), genus *flavivirus*, is antigenically classified into four serotypes (DENV1-4). DENV is an arbovirus, mainly transmitted by aedesaegypti mosquitoes. The virus is thought to be responsible for close to 400 million infections per year worldwide [2].

The World Health Organization (WHO) 2009 guidelines identified three diagnostic tests as golden standards for dengue diagnosis: viral isolation and identification, nucleotide detection, and serological tests for IgM or IgGseroconversion [3]. However, these methods have certain limitations. For example, viral isolation is difficult to perform and time-consuming. Nucleotide detection such as RT-PCR requires specialized laboratory equipments. And serological tests for IgM or IgG cannot be used for early onset diagnosis. Therefore, a more efficient and accurate detection method for DENV is warranted. Nonstructural protein 1 (NS1) is a glycoprotein that is abundantly produced by DENV in the early stage of infection, and can be detected in the serum [4, 5]. In contrast to IgM, which can only be detected at the beginning of the fifth day of the disease, NS1 antigen capture could be performed at the onset of symptoms.

Currently, several laboratory methods based on the capture of DENV NS1 antigen are available. The two main methods for detecting DENV infection are enzyme-linked immunosorbent assay (ELISA) (Platelia (Platelia Dengue NS1 Ag-ELISA Kit) and Panbio (Panbio Dengue Early ELISA Kit)) and immunochromatography (IC) (STRIP, Dengue NS1 Ag STRIP Kit). Previous studies have performed meta-analysis of the diagnostic accuracy of NS1 Ag-based tests including all published articles, however, there is still lack of evidence on the diagnostic accuracy of NS1 Ag-based tests in Asian population. Therefore, we conducted a meta-analysis to obtain the overall estimated and summarized performance of NS1 Ag-based tests in the detection of DENV in Asia.

## Methods

### Search strategy and inclusion criteria

A literature search was performed to screen studies in human that focused on the diagnostic performance of NS1 for the detection of Dengue. We searched PubMed, Medline and Embase database for relevant citations published in English from January 1990 to November 2014. The keywords “NS1” and “Dengue” were used. Two reviewers independently reviewed each publication. The abstracts were read to identify potentially eligible articles and then the full texts of these articles were examined to determine whether they should be included in our study. Any disagreement was discussed and solved by a third reviewer. The inclusion criteria were: (1) samples or patients with dengue confirmed by the standard methods including viral isolation and identification, RNA detection, or serological test for IgM and/or IgG; (2) samples or patients with dengue investigated by NS1 Ag-based capture methods; (3) have reported sufficient data to allow us to calculate the true positive (TP), false negative (FN), false positive (FP) and true negative (TN) values; (4) at least 20 samples from patients and control group, respectively; (5) the participants were Asian. Studies with an overlapping patients sample were excluded.

### Data extraction and quality assessment

The following information from each study was extracted independently by two reviewers: (1) first author name; (2) year of publication; (3) location of studies; (4) number of patients; (5) detection methods; (6) event numbers in TP, FN, TN and FP arms. QUADAS questionnaire was used to assess the quality of the studies included in this meta-analysis.

### Data analysis

The pooled sensitivity, specificity, diagnostic odds ratio (DOR) and the likelihood ratios (positive likelihood ratio (PLR) and negative likelihood ratio (NLR)) for single test were calculated by Meta-Disc version 1.4. The DOR was calculated as PLR/NLR. A hierarchical summarized receiver operating characteristic (HSROC) curve was plotted by the software. We also constructed the area under curve (AUC) that serves a global measure of the test performance.

The I-squared value (I^2^) was used to assess the statistical heterogeneity among the studies. The estimate below 25 % was regarded as low heterogeneity, while above 75 % was labeled as high heterogeneity [6]. If heterogeneity existed, a random effect model was used for meta-analysis; otherwise, a fixed effect model was chosen. Subgroup analyses were performed according to the detection methods (ELISA vs. IC), the manufacturers of the test kit, DENV serotypes, serological status, clinical severity and days after onset of fever to assess potential sources of variation in the study results.

In addition, a meta-regression was used according to the following pre-defined characteristics to explore the source of heterogeneity in the studies: study design, publication year, origin of sample, and sample size. A p-value of < 0.05 was considered to be statistically significant. To assess potential publication bias, the Deeks funnel plot asymmetry test was used, with *p* < 0.05 indicating the presence of publication bias [7]. Fagan nomograms, a two-dimensional graphical tool for estimating how much the result of a diagnostic test changes the probability that a patient has a disease, was used to estimate the clinical value of the index test.

## Results

### Literature search

**Fig. 1:**
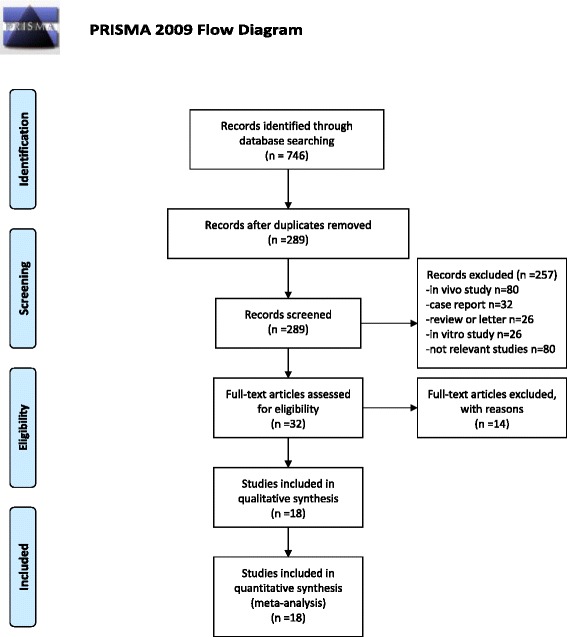
Flow diagram of the studies identified

A total of 143 citations were obtained via database searches, and eighteen met the inclusion criteria for this study (Fig. 1). These studies included 3342 dengue cases and 1904 control cases. Among the dengue cases, 439 patients had dengue hemorrhagic fever (DHF). 3129 samples were tested by Platelia Dengue NS1 Ag-ELISA Kit (Bio-Rad), 1081 samples by Early Dengue NS1 ELISA Kit (Pan-bio), and 1896 samples by Dengue NS1 Ag STRIP (Bio-Rad) (Table 1).

**Table 1:** Summary of the included studies

Study	year	country	age	Sex (M/ F)	Sample			Illness day	diagnosis	NS1 diagnosis
					DF	DHF	Control			
Hermann [8]	2014	Thailand	4–12	152/182	159	141	20	1–7	RT-PCR/ELISA	ELISA(Bio-Rad)
Gan [9]	2014	Singapore	18–68	193/53	147	NR	50	1–14	IgG/ELISA	ELISA(Bio-Rad)
Naz [10]	2014	Pakistan	2–65	96/88	142	NR	42	2–7	IgG/ELISA	Strip(Bio-Rad)
Aryati [11]	2013	Indonesia	NR	NR	188	NR	252	NR	VI/RT-PCR	ELISA(Panbio)
Huang [12]	2013	Taiwan	32–60	175/217	392	NR	50	1–8	VI/RT-PCR	Strip(Bio-Rad)
Kosasih [13]	2013	Indonesia	4–53	142/78	220	NR	55	1–7	RT-PCR/ELISA	ELISA(Bio-Rad)
Blacksell [14]	2012	Thailand	NR	NR	239	NR	50	NR	RT-PCR/ELISA	ELISA(Bio-Rad)
Watthanaworawita [15]	2011	Thailand	>15	98/64	72	NR	90	1–7	RT-PCR/ELISA	ELISA(Panbio)
Duong [16]	2011	Cambodia	4–11	203/136	101	87	139	3–8	VI/RT-PCR/ELISA	ELISA(Bio-Rad)
Chuansumrit [17]	2011	Thailand	<18	NR	19	36	30	2–10	VI/ ELISA	ELISA(Bio-Rad)/Strip(Bio-Rad)
Blacksell [18]	2011	Sri Lanka	NR	NR	239	NR	148	NR	RT-PCR/ELISA	ELISA(Bio-Rad, panbio)/Strip(Bio-Rad)
Fry [19]	2011	Vietnam	3–15	NR	198	NR	100	1–4	RT-PCR/ELISA	Strip(Bio-Rad)
Pok [20]	2010	Singapore	NR	NR	161	NR	160	1–8	RT-PCR/ELISA	Strip(Bio-Rad)
Hang [21]	2009	Vietnam	4–42	62–76	125	NR	13	1–10	RT-PCR/ELISA	ELISA(Panbio)
Zainah [22]	2009	Malaysia	NR	NR	100	NR	219	NR	VI/RT-PCR	Strip(Bio-Rad)
Blacksella [23]	2008	Vientiane	NR	NR	38	NR	54	NR	ELISA	ELISA(Panbio)
Lapphra [24]	2008	Thailand	3–52	132/103	151	19	64	1–5	VI/RT-PCR/ELISA	ELISA(Bio-Rad)
Kumarasamy [25]	2007	Malaysia	NR	NR	213	NR	368	NR	VI/RT-PCR	ELISA(Bio-Rad)

*NR* no report, *VI* virus isolation, *ELISA* enzyme linked immunosorbent assay, *DF* dengue fever, *DHF* dengue hemorrhagic fever

### Overall accuracy of the tests

The overall sensitivity and specificity of NS1 Ag-based test kits were 66 % (95 % CI 64.5–67.5) and 97.9 % (95 % CI 97.3–100), respectively. The sensitivity of NS1 Ag-based test kits in primary infection patients was 88 % (95 % CI 85.8–89.9) and in secondary infection patients was 60.8 % (95 % IC 57.8–63.8). According to the DENV serotypes, DENV1 had the highest sensitivity of 79.5 % (95 % CI 76.6–82.3) and DENV4 had the lowest sensitivity of 46 % (95 % CI 36–59.3). The AUC (Area Under ROC Curve) was 0.96. According to clinical severity and test assays, NS1 Ag-based test kits were more effective in diagnosing dengue fever (DF) than DHF, and IC had a higher sensitivity than ELISA (Table 2).

**Table 2:** Diagnostic accuracy results of overall NS1 Ag-based test kits

	No. of study	Sensitivity %	Specificity %	Heterogeneity %
		(95 % CI)	(95 % CI)	(I^2^)
Overall	18	66 (64.5–67.5)	97.9 (97.3–100)	82.6
Serological status				
Primary	9	88 (85.8–89.9)		89.2
Secondary	10	60.8 (57.8–63.8)		93.2
DENV serotypes				
DENV-1	10	79.5 (76.6–82.3)		81
DENV-2	11	62.7 (59.1–66.2)		84.2
DENV-3	10	73 (69.2–76.6)		79.5
DENV-4	5	46 (36–59.3)		56
Clinical severity				
Dengue Fever	5	70.3 (66.1–74.2)		80.8
Dengue Hemorrhagic Fever	5	58.8 (54–63.4)		71.6
NS1 test assays				
ELISA	14	63.3 (61.5–65.1)		94.9
IC	7	72.9 (70.1–75.5)		90.8

### Accuracy of the tests on the viral serotype, serological status and clinical severity

Among the included studies, eleven used Platelia, five usedPanbio and seven used Strip. For Platelia, the sensitivity and specificity were 66.2 % (95 % CI 64.1–68.2) and 97.3 % (95 % CI 96.1–98.2), respectively. The values for Panbio were 54 % (95 % CI 50.1–57.9) and 97.2 % (95 % CI 95.2–98.6), and the values for Strip were 72.9 % (95 % CI 70.1–75.5) and 99.1 % (95 % CI 98.2–99.6), respectively. The AUCs of Platelia, Panbio and Strip were 0.97, 0.96 and 0.98, respectively (Table 3).

**Table 3:** Diagnostic accuracy results of the NS1 Ag-based tests on the viral serotype, serological status and clinical severity

	ELISA			IC
	Platelia	Panbio	Overall	Strip
Overall				
No. of study	11	5	14	7
Sensitivity % (95 % CI)	66.2 (64.1–68.2)	54 (50.1–57.9)	63.3 (61.5–65.1)	72.9 (70.1–75.5)
Specificity % (95 % CI)	97.3 (96.1–98.2)	97.2 (95.2–98.6)	97.3 (96.3–98.1)	99.1 (98.2–99.6)
Heterogeneity % (I^2^)	84.1	79.8	82.4	40.5
DENV serotypes				
DENV1				
No. of study	7	2	8	3
Sensitivity % (95 % CI)	83.7 (79.7–87.1)	73.7 (65.5–80.9)	81.2 (77.7–84.4)	75.9 (70.1–81)
Heterogeneity % (I^2^)	82.5	89.6	84	57.8
DENV2				
No. of study	7	2	8	4
Sensitivity % (95 % CI)	56.9 (51.9–61.8)	71.8 (60.5–81.4)	59.3 (54.8–63.7)	69 (63–74.5)
Heterogeneity % (I^2^)	88.6	0	86.4	70.4
DENV3				
No. of study	6	2	7	4
Sensitivity % (95 % CI)	69.9 (64.8–74.6)	63.3 (48.3–76.6)	69.1 (64.3–73.5)	81.9 (75.5–87.2)
Heterogeneity % (I^2^)	82.3	15.3	76.9	75.5
DENV4				
No. of study	4	0	4	0
Sensitivity % (95 % CI)	42.9(32.1–54.1)		42.9 (32.1–54.1)	
Heterogeneity % (I^2^)	57.1		57.1	
Serological status				
Primary				
No. of study	7	2	8	2
Sensitivity % (95 % CI)	95.1 (92.6–96.9)	72.6 (65.6–78.9)	88.4 (85.7–90.8)	87.3 (83.6–90.5)
Heterogeneity % (I^2^)	56.8	29.2	89.1	94.6
Secondary				
No. of study	7	3	9	2
Sensitivity % (95 % CI)	63 (59.5–66.4)	50.8 (43.5–58)	60.6 (57.4–63.6)	64 (53.2–73.9)
Heterogeneity % (I^2^)	95.8	0	94.1	88.1
Clinical severity				
Dengue Fever				
No. of study	4	1	5	1
Sensitivity % (95 % CI)	72.1 (67.6–76.3)	51 (37–64)	69.5 (65.2–73.6)	89 (67–99)
Heterogeneity % (I^2^)	73.9		81.7	
Dengue Hemorrhagic Fever				
No. of study	4	1	5	1
Sensitivity % (95 % CI)	58.7 (52.7–64.5)	58.7 (49–67)	58.6 (53.6–63.4)	61 (43–77)
Heterogeneity % (I^2^)	82.9		77.2	

When evaluating the accuracy of these tests for DENV1, the sensitivity of Platelia was 83.7 % (95 % CI 79.7–87.1), while the value for Panbio was 73.7 % (95 % CI 65.5–80.9) and the value for Strip was 72.9 % (95 % CI 70.1–75.5). For DENV2, the pooled sensitivity was 56.9 % (95 % CI 51.9–61.8) for Platelia, 71.8 % (95 % CI 65.5–80.9) for Panbio and 69 % (95 % CI 63–74.5) for Strip. For DENV3, the pooled sensitivity was 69.9 % (95 % CI 64.8–74.6) for Platelia, 63.3 % (95 % CI 48.3–76.6) for Panbio and 81.9 % (95 % CI 75.5–87.2) for Strip. For DENV4, the pooled sensitivity was 42.9 (95 % CI 32.1–54.1) for Platelia (Table 3).

Regarding the classification of primary or secondary dengue infection, the sensitivities of Platelia, Panbio and Strip were 95.1 % (95 % CI 92.6–96.9), 72.6 % (95 % CI 65.6–78.9) and 87.3 % (95 % CI 83.6–90.5) for primary infection, and 63 % (95 % CI 59.5–66.4), 50.8 % (95 % CI 43.5–58) and 64 % (95%CI 53.2–73.9) for secondary infection, respectively (Table 3).

To verify whether such tests had significant variations in the performance in patients with DF or DHF, we performed subgroup analysis according to clinical severity, and the results showed that the pooled sensitivity of Platelia was 58.7 % (95 % CI 52.7–64.5) for DF, and 72.1 % (95 % CI 67.6–76.3) for DHF (Table 3).

### Accuracy of the tests on illness duration

We also evaluated the influence of the illness duration on the accuracy of the NS1 Ag-based tests. It was found that the sensitivity of the tests was higher in the first 3 days after the illness onset than in the following 4–7 days (Table 4).

**Table 4:** Diagnostic accuracy results of the NS1 Ag-based test on illness duration

	1–3 day			4–7 day		
	No. of study	Sensitivity % (95 % CI)	Heterogeneity % (I^2^)	No. of study	Sensitivity % (95 % CI)	Heterogeneity % (I^2^)
NS1	5	79.7 (75.2–83.8)	76.4	4	57.8 (53.3–62.3)	82.4

### Post-test probability and publication bias

**Fig. 2:**
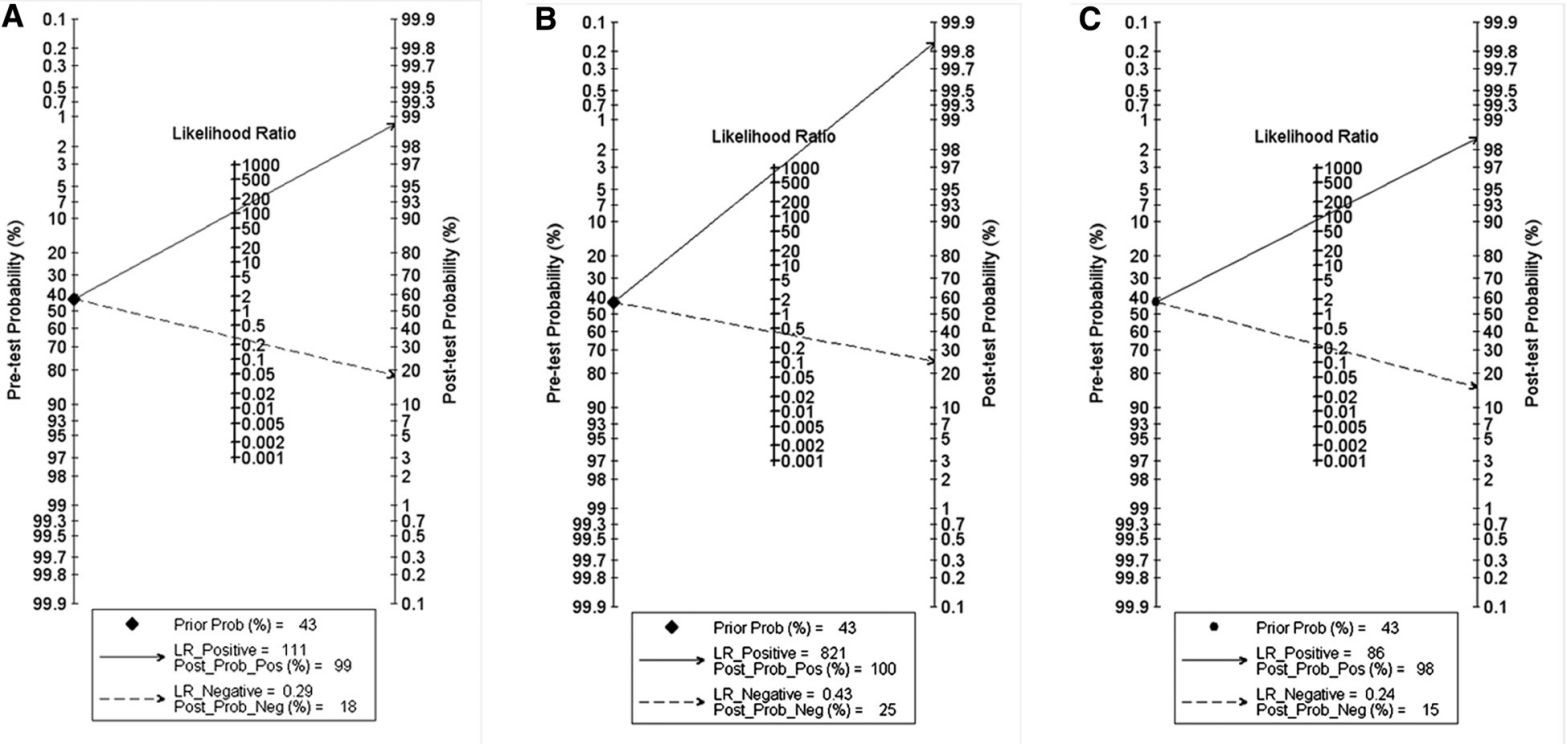
Fagan’s nomogram for the calculation of post-test probabilities. A pre-test probability of 42.9 % for dengue disease was fixed, which was estimated using the number of symptomatic cases in the selected studies. **a** Platelia had a post-test probability of 99 %. **b** For Panbio kits, post-test probability was 100 %. **c** Strip had a post-test probability of 98 %, ie, with an estimated prevalence of 37 %, if this patient tests positive, the post-test probability that he/she truly has dengue would be 98 % (solid line). On the other hand, if patient tests negative, the post-test probability that he/she truly has dengue would be 18 % (**a**), 25 % (**b**) or 15 % (**c**) (dotted line)

To obtain the post-test probability, we used Fagan’s nomogram to perform a simulation of an environment that had a prevalence of 42.9 % for dengue disease, an estimate that was generated based on the studies selected. Thus, the probability for someone that had the disease but was not detected by Platelia was 18 % in this model (Fig. 2a). Similarly, the value for Panbio was 25 %, and the value for Strip was 15 % (Fig. 2b-c). In contrast, the post-test probability of sick patient with a positive test result was 99 % for Platelia, 100 % for Panbio, and 98 % for Strip (Fig. 2a-c).

Additionally, the Deeks funnel plot did not show any potential publication bias for the subgroup studies (P_Platelia_ = 0.95, P_Panbio_ = 0.17 and P_Strip_ = 0.51).

## Discussion

The dramatic increase in Asian dengue burden has promoted social interest in improving dengue diagnosis. Although many dengue vaccines are under development, none has been licensed to date. Therefore, early diagnosis of dengue leads to appropriate clinical management and better outcome. Soluble NS1 detection in the serum or plasma of a DENV-infected patient is a novel method for early dengue diagnosis. To evaluate the accuracy of NS1 Ag-based test in dengue diagnosis, we performed a meta-analysis using published studies.

Our results indicated that NS1 Ag-based test has a high accuracy for dengue diagnosis. The AUC of NS1 Ag-based test was 0.96. The pooled specificity (97.9 %, 95 % CI 97.3–100) was extremely high, but the pooled sensitivity (66 %, 95 % CI 64.5–67.5) was relatively low. In this regard, NS1 Ag-based test may not be suitable to detect dengue as low sensitivity may result in misdiagnosis. Considering that the sensitivity of NS1 Ag-based test may be influenced by test method, viral serotype, serological status and clinical severity, we also performed subgroup analysis. According to the analysis of the different kits (Platebia, Panbioand STRIP), it appeared that Dengue NS1 Ag STRIP Kit had the higher sensitivity, followed by Platebia Dengue NS1 Ag-ELISA Kit.

Our meta-analysis showed that NS1 Ag-based test had a lower sensitivity for DENV4 and a higher sensitivity for DENV1 and DENV3. According to the analysis of the different kits, Platebia had the highest sensitivity for DENV1, Panbio had the highest sensitivity for DENV2, and STRIP had the highest sensitivity for DENV3. Unfortunately, the sensitivity data for DENV4 was not available for Panbio and STRIP, thus we were unable to compare the sensitivity of the three kits for DENV4. The reason for the wide range of total accuracy of serotyping for dengue may be due to the quantitative differences in the secreted NS1 by the different viral serotypes. However, this hypothesis needs to be confirmed by additional studies.

It was shown that the sensitivity of NS1 Ag-based test was higher for primary dengue infection than secondary infection. For patients with primary dengue infection, Platelia had the highest sensitivity. As for secondary infection, STRIP had the highest sensitivity. Detection of NS1 antigen in secondary infection may be hampered by a rapid rise in antibody levels due to the anamnestic antibody response [26], which leads to the formation of immune complexes and thus preventing the binding of capture or detection antibodies to NS1 antigen. However, simple and repeat hit of secondary DENV infected patients’ serums was shown to be very useful for increasing the sensitivity of the DENV NS1 Ag-based test. In addition, our results also revealed a small decrease of sensitivity of the NS1 Ag-based test for patients with DF compared to those with DHF. Moreover, the sensitivity of NS1 Ag-based test was higher in the first 3 days after illness onset than in the following 4–7 days, and again, the increased antibodies generated by the host’s immune response could be the reason.

There was significant heterogeneity in this meta-analysis and caution must be taken when interpreting the results. In addition, we have used meta-regression analysis to explore the potential factors that may be the source of the heterogeneity. Unfortunately, none of the covariates examined was the source of heterogeneity. Further studies were needed to identify the potential source for the heterogeneity.

There were also some limitations in our study. Firstly, the standard diagnosis methods of NS1 were different in the included studies, e.g., some studies used viral culture while others used serological test as standard method. Secondly, only a few authors used other types of fever as control samples, while most used samples from healthy persons as control. Thirdly, data were not divided into groups based on gender, age or other variables, due to the limited studies. Despite these limitations, we believe that our analysis could contribute to the evaluation of the accuracy of NS1 Ag-based test, which eventually might help the clinical decision making process.

## Conclusion

Our meta-analysis suggests that NS1 Ag-based test was a good diagnosis method for dengue with a high specificity. However, viral serotype, serological status, clinical severity and illness duration were the main factors influencing the diagnosis accuracy. Moreover, the different test kits have their own advantages/disadvantages. Large, multicenter prospective studies are needed to confirm our results.
